# Care of Older Persons in Eastern Africa: A Scoping Review of Ethical Issues

**DOI:** 10.3389/fpubh.2022.923097

**Published:** 2022-07-06

**Authors:** Kirubel Manyazewal Mussie, Jenny Setchell, Bernice Simone Elger, Mirgissa Kaba, Solomon Tessema Memirie, Tenzin Wangmo

**Affiliations:** ^1^Institute for Biomedical Ethics, University of Basel, Basel, Switzerland; ^2^School of Health and Rehabilitation Sciences, The University of Queensland, Brisbane, QLD, Australia; ^3^Center for Legal Medicine, University of Geneva, Geneva, Switzerland; ^4^School of Public Health, Addis Ababa University, Addis Ababa, Ethiopia; ^5^Addis Centre for Ethics and Priority Setting, Addis Ababa University, Addis Ababa, Ethiopia; ^6^Harvard T.H. Chan School of Public Health, Harvard University, Boston, MA, United States

**Keywords:** care, health care access, isolation, gender, elder abuse, scoping review

## Abstract

**Introduction:**

The aging population is rapidly increasing globally, with 80% of the older population living in low- and middle-income countries. In Eastern African countries, there exists an incongruence between readiness–economically, structurally, politically, and culturally–to create a conducive environment for healthy aging, which implies public health as well as ethical concerns. The aim of this scoping review was to explore existing evidence addressing the various ethical issues in connection with elder care in the region of Eastern Africa.

**Methods:**

We searched six databases (Africa-Wide Information, AgeLine, CINHAL, MEDLINE, APA PsycInfo, and SocINDEX) to identify peer-reviewed journal articles that could meet some eligibility criteria such as being a peer-reviewed journal article written in English, having been published in any year until July 2020, and focusing on ethical issues in the care of older people aged 60 years and older from Eastern Africa. We also searched for additional evidence in the references of included papers and web-based platforms. We included 24 journal articles and analyzed them using the inductive content analysis approach.

**Results:**

The included articles represent seven (38.9%) of the 18 countries in the Eastern African region. The articles covered six ethical concerns: lack of government attention to older persons (*n* = 14, 58.3%), inaccessibility of health care services (*n* = 13, 54.2%), loneliness and isolation (*n* = 11, 45.8%), gender inequalities in old age (*n* = 9, 37.5%), mistreatment and victimization (*n* = 8, 33.3%), and medical errors (*n* = 2, 8.3%).

**Conclusion:**

This scoping review summarized ethical issues arising in relation to providing care for older persons in the Eastern African context. In light of the rapid increase in the number of older persons in this region, it is critical for governments and responsible bodies to implement and accelerate efforts promptly to generate more evidence to inform programs and policies that improve the health and wellbeing of older persons. Further research is needed to inform global health efforts that aim at improving the lives of older persons, particularly in low- and middle-income countries.

**Clinical Trial Registration:**

https://osf.io/sb8gw, identifier: 10.17605/OSF.IO/SB8GW.

## Introduction

The world's older population (≥60 years as defined by the UN (1)) is showing a dramatic increase: it was 900 million in 2015 and is expected to total two billion by 2050 (2). Of this projected demographic, 80% concerns low- and middle-income countries (2). In sub-Saharan Africa only, the number of older people (≥60 years) is projected to reach 163 million by 2050 (3). Older people in this region experience high levels of both infectious and non-communicable diseases–the latter being the main cause of death for many older adults in the least developed countries (4). In a study conducted among older patients in sub-Saharan African countries, non-communicable diseases were accountable for 81% of hospital admissions for older patients in Nigeria, Sudan, and Tanzania (5). “Poor health does not have to be the dominant and limiting feature of older populations” (6) and thus, the determinants of poor health among older adults in low-income countries warrant much attention and further research.

Very little attention has been given to aging research in Sub-Saharan Africa despite the rapidly growing number of older adults and their important role in communities, for example, in taking care of younger kin (7). Care needs of older people in Africa, in general, are higher than older persons in other continents such as Europe. For example, in South Africa, more than 45% of people aged 75 and older require assistance with daily activities, while 20% of the same age group in Switzerland require assistance (8).

Generally, care for older persons in Eastern Africa is not well-developed and is characterized by complexities. The growing number of the older population in the region, combined with rapid social and economic changes, brings more complex questions about care and caregiving for older persons. Although there are strong traditional family and community support for older people in the region, family support dwindles when a caregiver, for example, dies or migrates to cities or other countries in search of better job opportunities (9, 10). As one review suggests (11), “industrialization, urbanization, and globalization have caused young people to leave the farms for city, leaving their elderly parents and traditions behind” and this causes ethical concerns such as loneliness and isolation of older people. On the other side, existing formal health system structures can have limitations to accommodate the health needs of all population groups such as older adults (12, 13).

Thus, the purpose of this scoping review is twofold. First, as shown above, there are particular conditions that imply a compromise in the health and wellbeing of older adults in the region. This situation raises ethical concerns such as, for example, health care access, distributive justice, and questions such as who is responsible for caring for older adults, and how should their rights at old age be protected. The study of ethical issues in old age care in this region is hence necessary to inform both policy and practice that can improve the health and wellbeing of older persons in Eastern Africa. Second, there have been no studies to date reviewing ethical concerns in aged care in the region. A pilot search conducted on 13 June 2020 indicated that there were neither protocols nor finalized reports of systematic or scoping reviews on ethical issues in old age care in Eastern Africa. The most relevant papers that we could identify after searching five databases–African-Wide Information, AgeLine, CINAHL, MEDLINE, and Google Scholar–include one systematic review protocol on the effectiveness of interventions for dementia in low- and middle-income countries (14), one systematic review of qualitative studies on end-of-life care in sub-Saharan Africa (15), and one protocol for a scoping review of age-related health conditions among geriatric populations in sub-Saharan Africa (16). However, none of these articles specifically focussed on ethical aspects of elder care. This gap shows that ethical issues in aged care in Eastern Africa warrant further exploration. The purpose of this scoping review was to explore existing evidence addressing the various ethical issues in connection with elder care in the region of Eastern Africa, which consists of the following 18 countries according to the UN Statistics Division: Burundi, Comoros, Djibouti, Eritrea, Ethiopia, Kenya, Madagascar, Malawi, Mauritius, Mozambique, Réunion, Rwanda, Seychelles, Somalia, Uganda, United Republic of Tanzania, Zambia and Zimbabwe (17). It is anticipated that the results of this scoping review will inform governments and policymakers of the ethical issues in old age care in Eastern Africa.

## Methods

We conducted a scoping review of the literature to capture evidence about ethical issues in the care of older people in Eastern Africa who are 60 years old or above. The United Nations agreed to refer to geriatric populations as those who are 60 and above in Africa whereas age 65 is used to define geriatric populations in high-income countries (1). Scoping reviews are conducted mainly to identify the types of available evidence on emerging topics and to analyze the existing knowledge base (18). Therefore, this methodology proves suitable for this work considering our broad research question which aims at addressing ethical issues in aged care in Eastern Africa. We applied Arksey and O'Malley's scoping review framework consisting of five stages: identifying the research question, identifying relevant studies, study selection, charting the data, and collating, summarizing, and reporting the results (19). In addition, we used the PRISMA checklist for reporting scoping reviews (20). Six reviewers were involved in this scoping review to reduce errors and increase reliability in the review process. The scoping review complies with what Peters et al. state: “as with all systematic reviews, *an apriori* scoping review protocol must be developed before undertaking the review itself” (21). Moreover, the review was registered in OSF (Open Science Framework) on 04 January 2021 (registration https://doi.org/10.17605/OSF.IO/SB8GW) in compliance with the recommendations given in JBI (Joanna Briggs Institute) Manual for Evidence Synthesis (22).

### Literature Search

**Figure 1:**
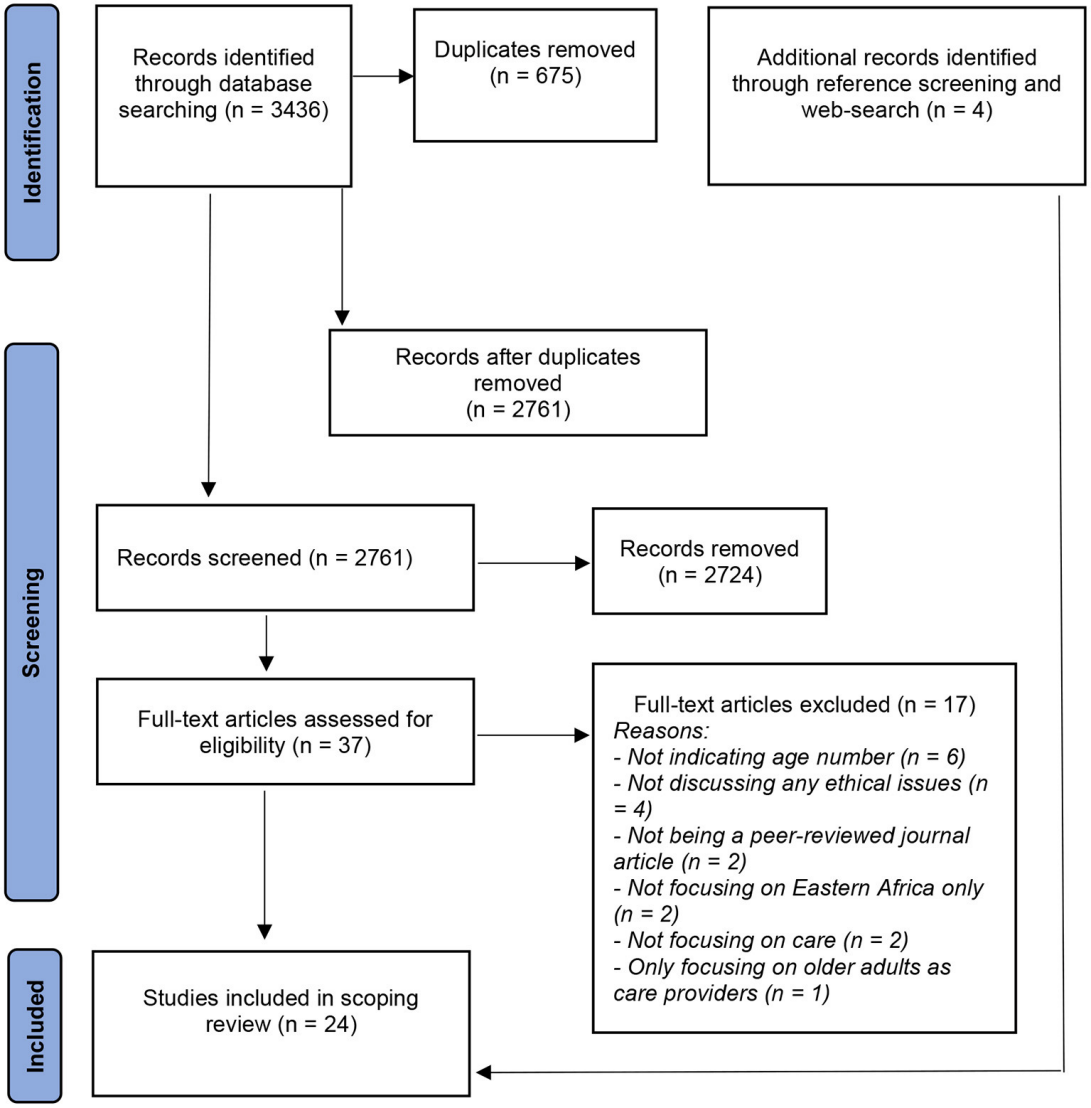
PRISMA flow diagram.

The search strategy followed the three-step method recommended in standard JBI systematic reviews (23). Moreover, we followed a rigorous search strategy with support from an experienced librarian to clarify our search strategy (19). First, we undertook a preliminary search of selected databases (MEDLINE and SCOPUS) and analyzed index terms and text words contained in titles and abstracts. We gathered and identified the keywords by searching MeSH (Medical Subject Headings) terms and subject headings in the databases, searching on the internet (e.g., Google Scholar), and incorporating expert comments. We broke down our research question–what are the ethical issues in the care of older persons in Eastern Africa–into broad keywords and MeSH terms such as “older people” or “older adults” or “senior citizens” and “east Africa.” We developed a search strategy for each database with a combination of free text and controlled vocabulary. Our strategy in developing and using search keywords was kept broad and flexible to increase the chances of getting relevant papers. Second, we used all identified keywords and index terms to search the following six databases: Africa-Wide Information, AgeLine, CINHAL, MEDLINE, APA PsycInfo, and SocINDEX. The search strategies used for these databases can be found in Additional File 1. This search, conducted on 21^st^ and 22^nd^ July 2020, yielded 3436 results (Figure 1). Third, the reference list of all included papers was searched for additional evidence. At that stage, we also searched additional web-based platforms such as Google in June 2021 to identify more and recent literature.

### Eligibility Criteria

The inclusion criteria for the articles were as follows: written in English, peer-reviewed journal articles, published in any year until July 2020, focused on people aged 60 years and older from Eastern Africa, addressed ethical issues in connection with how older adults are treated in communities and/or health care settings, and focused on one, some or all of Eastern African countries.

### Study Selection

The evidence selection process followed the Preferred Reporting Items for Systematic Reviews and Meta-Analyses Extension for Scoping Reviews (PRISMA-ScR) checklist (20). First, all the 3,436 articles that the search yielded were uploaded into Endnote X9 software. Then the first author (KMM) identified and removed 675 duplicates. Two of the authors (KMM, TW) independently screened the abstracts and titles of the remaining 2,761 articles to identify and exclude those papers that did not meet the eligibility criteria. Any disagreements regarding the study eligibility of the selected articles were discussed between the two reviewers until a consensus was reached. This screening resulted in 37 articles that were distributed between four reviewers (JS, BE, MK, STM) for full-text review. After the full-text review, 17 articles did not meet one or more of the eligibility criteria for inclusion and thus were removed. The criterion given more weight at this stage was whether the issues the papers deal with have ethical implications. The remaining 20 articles were selected for the review stage. As stated earlier, four additional articles were added during the review by checking, for example, the references of the included studies. The last 4 articles were reviewed and included by KMM, thereby making the final number of articles included in this study 24.

### Data Charting

Metadata from the articles were extracted using a proforma (Table 1) the first author prepared, which was reviewed by part of the team when the review protocol was prepared. The charting form was kept flexible and was updated as necessary throughout the charting process.

**Table 1:** Categories of information extracted from the articles.

**Author/s, date and title**
Publication type
Country/location of study
Objective
Design and methods
Sample (study population)
Main findings
Recommendations for future research (if any)
REC/IRB approval

### Collating, Summarizing, and Reporting the Results

The MAXQDA software was used to analyze the articles (24). Using the inductive content analysis approach (25) and with inputs from TW, KMM openly coded the included papers using the analysis software (MAXQDA 2020) to identify recurring codes. The codes were then grouped into broader themes and all authors discussed their relevance as ethical issue, and refined and reconstructed the themes wherever necessary. A summary of the justification for why the themes were considered ethically relevant is provided in the Additional File 2. Based on comments from all authors, KMM reviewed the initial codes and related contents of the articles and revised the themes accordingly to confirm that no ethically relevant aspects were missed. The analysis resulted in six ethically relevant themes collated from the included manuscripts.

## Results

### General Description of the Articles

A total of 24 articles were included in this scoping review. Table 2 summarizes the general characteristics of the included articles.

**Table 2:** Overview of the included studies.

**Author/s and reference number**	**Publication type**	**Countries**	**Objective (s)**	**Study design**	**Study population**	**Distribution of themes over included articles**	**REC/IRB approval^**g**^**
Abegaz et al. (26)	Original research article	Ethiopia	Assess potentially inappropriate prescriptions and associated factors in the older people with cardiovascular disorders using the START/STOPP screening criteria.	Cross-sectional study using a structured questionnaire	239 Older patients aged 65 years or older with cardiovascular disorders	Medical errors	Yes
Ajwang et al. (27)	Original research article	Uganda	Explore the geriatrics continuing education needs of health care providers (HCPs) working in rural Uganda	Quantitative study using a self-administered questionnaire	240 Health care professionals working with older adults aged 60 and above	Inaccessibility of health care services	Yes
Allain et al. (28)	Original research article	Zimbabwe	Record the prevalence of disability (impairment of activities of daily living), subjective morbidity (symptoms), the social circumstances and the utilization of health services in a group of older Zimbabweans.	Cross-sectional community survey	278 Older people aged 60 and above	Inaccessibility of health care services; lack of government attention to older persons	Yes
Chane and Adamek (29)	Original research article	Ethiopia	Examine the types and nature of abuse and neglect from the perspective of elders in Ethiopia who experienced abuse in non-institutional settings	Qualitative phenomenological study using interviews	15 Older people aged 64 and above	Mistreatment and victimization; lack of government attention to older persons	Not reported^f^
Chane and Adamek (30)	Original research article	Ethiopia	Increase understanding of elder abuse in Ethiopia by considering the perspectives of abused elders.	Qualitative phenomenological study using interviews	15 Older people aged 64 and above	Loneliness and isolation; mistreatment and victimization; lack of government attention to older persons	Not reported
Dhemba (31)	Literature review	Zimbabwe	Explore the factors associated with the syndrome of poverty in old age in developing countries in general and Zimbabwe in particular.	A review of theoretical and empirical literature including policy documents	Older people aged 60 and above^e^	Lack of government attention to older persons	Not applicable
Getachew et al. (32)	Original research article	Ethiopia	Assess the prevalence of inappropriate prescribing of antithrombotic therapy in hospitalized older patients.	Retrospective cross-sectional study using review of patients' medical records	156 Hospitalized older patients aged 65 and above	Medical errors	Yes
Golaz and Rutaremwa (33)	Original research article	Uganda	Contribute to the understanding of vulnerability among the older population by using a major data source – the population census – and provide basic results concerning the vulnerability of older people for the case of Uganda.	Review of national census data.	Older people aged 60 and above ^e^	Inaccessibility of health care services; lack of government attention to older persons; gender inequalities in old age	Not reported
Golaz et al. (34)	Original research article	Uganda	Understand vulnerability among older adults in Uganda.	Qualitative study using in-depth interviews	Older persons aged 60 and above and their relatives (83 participants in total^b^)	Loneliness and isolation; inaccessibility of health care services; lack of government attention to older persons	Yes
Kakongi et al. (35)	Original research article	Uganda	Explore pathways to hospital care for patients with Alzheimer's disease and related dementias, and describe challenges experienced by the patients and their families while seeking health care.	Qualitative study using in-depth interviews	30 Caregivers of older adults aged 60 and above who were diagnosed with dementia	Inaccessibility of health care services	Yes
Kibuga and Dianga (36)	Original research article	Tanzania	Examine reasons for the killing of older women suspected of being witches.	Qualitative study using individual interviews and group discussions	Older persons aged 60 and above; caregivers and the families of older persons aged 60 and above; younger persons; village government leaders and religious leaders; traditional healers and birth attendants; and professionals (teachers, medical practitioners, etc)^a^	Loneliness and isolation; mistreatment and victimization; inaccessibility of health care services; lack of government attention to older persons; gender inequalities in old age	Not reported
Malambo and Marais (37)	Original research article	Zambia	Identify the barriers to the utilization of physiotherapy services among older people in Zambia.	A quantitative cross-sectional study using a self-administered questionnaire	200 Older adults aged 60 and above	Mistreatment and victimization; inaccessibility of health care services	Not reported
Mapoma and Masaiti (38)	Original research article	Zambia	Investigate the extent to which social-demographic variables impact risk factors associated with social isolation among older people in Zambia.	Quantitative study using a structured questionnaire	690 older people aged 60 and above	Loneliness and isolation; gender inequalities in old age	Not reported
Mushi et al. (39)	Original research article	Tanzania	Examine the socio-cultural beliefs surrounding dementia and the life experience of people with dementia and their caregivers in the Hai District of Kilimanjaro, Tanzania.	Cross-sectional qualitative design using in-depth interviews	41 Participants in total: 25 older people aged 70 and above with dementia and 16 caregivers	Lack of government attention to older persons	Yes
Nzabona et al. (40)	Original research article	Uganda	Investigate prevalence and correlates of feeling lonely among older persons.	Mixed methods study using an interviewer-administered questionnaire, focus group discussions, and individual interviews	697 Participants in total: 685 older persons (60 and above) and 12 key informants	Loneliness and isolation	Yes
Richards et al. (41)	Original research article	Uganda	Explore how women's and men's gendered experiences from childhood to old age have shaped their vulnerability in relation to HIV both in terms of their individual risk of HIV and their access to and experiences of HIV services.	Qualitative study using in-depth interviews and focus group discussions	38 Participants in total: 31 older persons aged 60 and above and 7 key informants (including local leaders and health workers)	Loneliness and isolation; mistreatment and victimization; inaccessibility of health care services; lack of government attention to older persons; gender inequalities in old age	Yes
Sadruddin (42)	Original research article	Rwanda	Explore how older Rwandans from different ethnic and gender groups provide and receive care from each other in the wake of sweeping social, economic, and demographic transformations post-genocide.	Ethnographic study using interviews and field observation	Older people aged 60 and above, caregivers, healthcare professionals, government officials, and local academics^a^	Loneliness and isolation; lack of government attention to older persons; gender inequalities in old age	Yes
Schatz et al. (43)	Original research article	Uganda	Explore the factors that cause older Ugandans (60+) to delay seeking, reaching and acquiring health care.	Qualitative study using interviews and focus group discussions	Older people aged 60 and above and key informants (health care providers and community leaders)^d^	Mistreatment and victimization; inaccessibility of health care services; lack of government attention to older persons; gender inequalities in old age	Not reported
Tam and Yap (3)	Original research article	Uganda	Examine the multidimensional challenges that an older woman with HIV in rural Uganda faces, and make contextualized policy recommendations for older adults in Africa.	Case study	One 70-year-old widow diagnosed with HIV	Loneliness and isolation; inaccessibility of health care services; gender inequalities in old age	Yes
Tegegn et al. (44)	Original research article	Ethiopia	Assess the medication-related quality of life (MRQOL) among older patients with polypharmacy at Gondar University Hospital, Gondar, Ethiopia.	Cross-sectional quantitative survey	150 Older patients aged 65 and above	Inaccessibility of health care services	Yes
Teka and Adamek (10)	Original research article	Ethiopia	Examine staff and resident perceptions of the psychosocial needs of elders in institutional care in Ethiopia and current efforts to provide psychosocial support.	Qualitative study using document review, field observation, interviews, and focus group discussions	29 Participants in total: 24 older adults aged 60 and above and 5 key informants (staff at the institution)	Loneliness and isolation; inaccessibility of health care services	Not reported^c^
Waweru et al. (45)	Original research article	Kenya	Determine the health status and the health-seeking behavior of older people.	Mixed study: descriptive cross-sectional methods using questionnaires and qualitative methods using focus groups discussions	400 Older persons aged 65 and above	Inaccessibility of health care services; lack of government attention to older persons	Not reported
Wright Set al. (46)	Original research article	Uganda	Explore despondency and psychological the wellbeing of older people.	Qualitative study using interviews	26 HIV-positive older persons aged 60 and above	Loneliness and isolation; mistreatment and victimization; lack of government attention to older persons; gender inequalities in old age	Yes
Zelalem et al. (47)	Original research article	Ethiopia	Explore experiences of family support among older adults in agrarian communities with a focus on filial responsibility expectations and intergenerational relations.	Qualitative phenomenological study using in-depth interviews	10 Older people aged 70 and above	Loneliness and isolation; mistreatment and victimization; lack of government attention to older persons; gender inequalities in old age	Yes

^a^*The study does not provide information regarding the number of participants*.

^b^*The study does not specify the number of participants from each participant group separately*.

^c^*The authors obtained a support letter from local authorities (Oromiya Labor and Social Affairs Office) to conduct the study. However, no information is given if this letter was obtained after an ethical review*.

^d^*The study does not clearly mention the number of participants. However, it notes that each of the nine focus group discussions had seven or eight participants. Moreover, it mentions that nine interviews were conducted with health care workers and community leaders*.

^e^*The review focuses on people aged 60 years and older. However, there is no further information about this population group except that data were taken from the national census*.

^f^*The study does not clearly state if ethical approval was obtained. But it generally mentions that it followed ethical procedures to protect participants*.

^g^*This column is used to indicate whether the study obtained ethical clearance from the relevant REC (research ethics committee) or IRB (institutional review board)*.

The majority of the studies were conducted in Uganda (*n* = 9) and Ethiopia (*n* = 7), covering respectively, 37.5 and 31.8% of the studies included in this scoping review. In terms of study design and methodology, a majority of the studies (*n* = 13) used qualitative study design employing either interviews or focus group discussions. The remaining articles used methods such as quantitative surveys and cross-sectional interviews (*n* = 7), mixed methods of quantitative and qualitative approaches (*n* = 2), and review (*n* = 2). Of the 23 studies that used primary data, 14 focused on older persons only while eight studies involved both older people and other participants such as informal caregivers, health professionals, and government officials. One study (27) included only health professionals working with older adults aged 60 and above. The remaining article (31)–which is a literature review–only focused on older persons.

Of the 23 original research studies, 14 indicated that they received ethics approval from concerned ethics review committees. One study (10) indicated that only a support letter was obtained from a government authority and does not indicate if this body reviewed and approved the study. An additional study (29) included a general statement concerning ethics approval: “To safeguard the elders, ethical procedures were followed throughout the research process.” The remaining studies (*n* = 7) did not provide any information regarding ethical review or approval.

### Ethically Relevant Issues

The below sections present the findings from our thematic analysis of the content of the included articles in relation to our research question. There were six themes coded: (1) loneliness and isolation, (2) mistreatment and victimization, (3) inaccessibility of health care services, (4) medical errors, (5) lack of government attention to older persons, and (6) gender inequalities in old age.

#### Loneliness and Isolation

Eleven of the included studies discuss loneliness and isolation of older persons, including their prevalence, factors, and consequences on the lives of older adults. In one quantitative study involving 690 older adults, three among five of the participants reported a feeling of loneliness (38). This proportion is higher in another, mixed study conducted among 685 older adults in which seven out of ten older adults reported loneliness (40).

Ten included studies further discuss the factors that contributed to older peoples' loneliness and social isolation. They mostly present modernization and the erosion of traditional African cultural values as the main contributors to the degradation of eldercare in Eastern Africa. “Changing global trends have eroded traditional African values of family solidarity” (3) and this has led older Eastern Africans to experience, among other things, loneliness and lack of attention, particularly from family members (34, 47). These challenges were not present in the past at the same intensity they currently exist. That is, the past was often characterized by family members having the will and time to care for their older relatives whereas in recent times, the dignity attached to aging weakened, and the attention and care older adults once used to receive grew weaker (30, 47). In addition to the cultural change, another way modernization and industrialization were argued to negatively impact the lives of older Eastern Africans was the emergence of new employment trends and opportunities that occupied the younger generation. The suggestion was that the emotional and physical care needs of older people were being left unmet when younger persons were busy meeting growing pressures to attend education and migrate for employment (34, 41, 42). As a result, as one study reports, for example, “elders were discontent with the support from their adult children, as the support was inadequate in light of their former filial responsibility and intergenerational cohesion” (47). The following quote from another study with older persons in Tanzania further elaborates this idea:

“If the older members fell ill, there would always be someone to look after them. Nowadays many older Africans report that their adult children have left the villages because of economic pressures: land is becoming scarcer, and in the new economic climate of cost sharing in Tanzania, it is more difficult to earn enough income through agriculture to pay for school fees and medical treatment than through work in the cities. Now, older members of a family tend to remain in the village and often end up living alone” (36).

#### Mistreatment and Victimization

Eight of the included studies discuss the mistreatment and victimization that older adults in Eastern Africa experience in both community and health care settings. Studies note that many elders were humiliated by the youth in general in their communities (30, 47). For example, one study (30) from Ethiopia reports that older persons experience verbal insults from some community members “because of their style of dress, slack posture, and physical appearance.” Whereas in Tanzania, as one study (36) on the victimization and killing of older women reports, the “witchcraft” label attached to aging (or a belief that older persons often practice witchcraft) “can lead to the isolation of older persons, particularly women, and in extreme but increasingly common instances, to murder.”

The mistreatment of older persons in communities at large also extends to health care settings in particular. Four studies (36, 37, 41, 43) explicitly report that older persons sometimes experience mistreatment by health professionals in health facilities. For instance, a study conducted in Uganda reports that “health workers shouted, were rude, accused older people of wasting their time and taking medicine that should go to younger persons, and criticized older people for not hearing, understanding or acting “properly” (43). Such health professional misconduct is likely to affect older people's health-seeking behavior, as indicated in another study that notes that mistreatment from health professionals is one of the factors that discourage older adults from seeking physiotherapy services (37). Instead of visiting healthcare facilities, older persons prefer to use alternative healthcare services. As one paper explains, “traditional healers are more accessible and familiar, and they perceptibly treat older clients with greater respect than do orthodox health professionals” (36).

#### Inaccessibility of Health Care Services

Thirteen publications report on the inaccessibility of health services and the consequences thereof on the health of older adults. As compared to other population groups, older adults have less access to health care facilities (33). For example, one study (37) reports that the majority (61%) of the older adults who participated in the study were unable to access physiotherapy services. Other studies identify some reasons for this problem. One of the reasons given was the unaffordability of health care services for older people: their access is limited by “lack of insurance or money to meet treatment bills (35). As one study reports (35), some older patients and their families sell their assets to pay medical expenses. To escape costs, some older persons go to local traditional healers who they believe have “negotiable fees” or they remain at home praying and exercising other religious practices (3, 35, 36). Others reported buying medications without prescriptions and formal medical consultation to avoid care expenses, thereby likely increasing their vulnerability to medical complications and additional diseases (45).

A second reason affecting older adults' access to health services is possibly linked with the inadequacy of health care facilities to meet the health needs of older patients. For example, one study highlight that “older women and men are a forgotten group since interventions are mainly designed for children below five and lactating mothers” (41). Specialist care services for diseases common in old age–such as Alzheimer's disease or related dementias–are limited (35, 45). An additional challenge the included articles discuss is limited geriatric knowledge among health professionals, which is reported to affect the quality of health care services older patients receive. As one study reports, “older persons see some of their inability to access quality care as connected to the lack of health-care workers with geriatric specialty training” (43). Another study adds:

“Currently, good health care for OAs [older adults] living in rural Uganda is difficult to achieve because, as shown by the findings of this study, the majority of HCPs [health professionals] who take care of OAs have a poor or fair geriatric knowledge and were educated on curricula that did not include geriatric content” (27).

Thus, even when local governments subsidize treatment costs to address older patients' financial burden, care services older patients receive can at times be of insufficient quality with potential effects of reducing healthcare-seeking behavior (43). Moreover, due to their expensive and demanding nature, treatments for non-communicable diseases such as diabetes and hypertension are not readily available for older patients. For instance, “health centers do not stock the appropriate drugs for diseases that specifically attack older people (such as diabetes, hypertension and arthritis) since they are expensive” (41). Elaborating the inadequacy of health facilities, one study adds that diagnostic instruments are scarce or unavailable and additionally highlights the scarcity of basic materials in long-term care facilities for older people (10).

“The older adult care center in Ethiopia was very resource poor and lacked even basic amenities such as soap and toilet tissue. The destitute circumstances impacted residents' social and psychological well-being both directly and indirectly. Several residents indicated dissatisfaction with the provision of the same items of food every day” (10).

A third reason affecting older persons' access to health care is the geographic location of health care facilities. Six studies (3, 10, 28, 36, 37, 43) report that the location of the health facilities is not convenient for older patients. Since dispensaries are usually located at a long distance from places where older persons reside, frail older adults with mobility issues do not receive medical assessments (10, 36). In order not to face the long distances to health facilities, older persons sometimes choose to wait until they experience severe health conditions or symptoms, which “warrant spending the money or social capital for transportation to the health center” (43). One study among physically impaired older Zimbabweans elaborates the situation as follows:

“Most subjects stated they would choose to visit their local clinic if they were taken ill, however, a number could not get there, or only with assistance. The ability to get to the clinic declined with age. The village health worker system did not seem to be adequately addressing this problem. Although those living in the townships lived closer to their local clinic than those in the rural areas the same percentages had difficulty getting to the clinic in both environments. This was despite the fact that more of the urban elderly were taking medication” (28).

#### Medical Errors

Two studies (26, 32), both from Ethiopia, report on medical errors and their factors. One of the studies (32), for instance, reports that there were a significant number of older patients exposed to inappropriate prescriptions at the participating health facility. Similarly, another study (26) reports that “potentially inappropriate prescription was higher among older cardiovascular patients” and that, for example, “ACEIs [Angiotensin-converting enzyme inhibitor] were the most commonly mis-prescribed medications.” A major factor the studies highlight for inappropriate prescriptions among Ethiopian older patients is longer hospital stays. Longer hospital stays cause more (iatrogenic) diseases among older patients and this leads to more medication, thereby increasing the chance for inappropriate prescriptions (26). There is insufficient information in the studies regarding the causes of longer hospitalization for these patients.

#### Lack of Government Attention to Older Persons

Fourteen studies mention two larger social factors that have ethical implications on the care of older adults in Eastern Africa. The first factor is weak legal support to protect older adults from abuse and mistreatment. For example, in some Eastern African communities where there are traditional practices of killing older women accused of witchcraft, government authorities are reported to show a lack of interest in protecting this population group. As one study (36) conducted in rural Tanzania reports, community members who reported “witchcraft” killings to village governments accuse them of “turning a blind eye to *uchawi* [witchcraft] killings, hence encouraging the practice.” Another study (30) from Ethiopia also reports a similar challenge: “Currently, the government in Ethiopia does not provide any of these protections [free legal services for abused elders, to protect and return their property, and to enforce wills]. There are few, if any, formal efforts to prevent elder abuse at any level.”

The second factor some studies highlight is the lack of attention from governments to improve the wellbeing of older adults. This is manifested in different ways, of which one is the lack of social security schemes for older adults. In some cases, older adults are not included in existing social security schemes that cover other population groups. For example, “available data show that the majority of older persons in Zimbabwe are not covered by existing social security schemes” (31). Another is low government spending on geriatric care, where a study (45) conducted in Kenya reports, “the decline in government funding of health services has affected health care delivery system” and as a result, “there are no special health services for geriatrics in Kenya.”

#### Gender Inequalities in Old Age

We identified nine studies that report inequalities between older men and women and the resulting vulnerabilities of older women. In addition to the challenges that come with aging, older women in some communities are vulnerable to additional challenges because of their gender. For example, in Tanzania, “older Tanzanian women face a double discrimination because of gender and age” (36). Most of these problems are connected to cultural beliefs that harm women. For example, according to one study conducted in rural Tanzania, the option of remarriage is seldom open to widowed older women, unlike older men due to beliefs that discourage the remarriage of women (36). This not only leaves older women lonelier and more isolated than older men (33, 38), but it also increases their vulnerability to health conditions such as depression (46). An additional component is that women might have difficulty accessing health care due to their gender: “women may be more likely to lack the types of social network that can be tapped for help to reach health facilities” (43).

## Discussion

This scoping review adds useful evidence to better understand the ethical landscape associated with elder care in Eastern Africa. More specifically, it sheds light on the ethical aspects of isolation, mistreatment, limited access to health care services, medication error, limited government commitment, and gender dynamics among older persons in Eastern Africa. There were substantial differences concerning the depth at which each theme was discussed, with health care access and government commitment being the most discussed and medical errors the least.

One finding of this scoping review is loneliness and social isolation among older persons in Eastern Africa. The main reason for this is the changes brought by modernization. The increasing prevalence of loneliness among older people in the region can be linked to the growth of globalization and cultural changes (3, 34, 47). The increasing trend of loneliness among older people in Eastern Africa is worrying from an ethical point of view. This is likely due to, among other reasons, the strong connection between loneliness and ill health in older adults (48). Further expanding this discussion, Lederman (49) mentions the following three reasons why loneliness is an ethical concern: it causes serious health problems, it is becoming significant on the clinical and public health agenda, and “it engenders several ethical and philosophical questions as a social determinant of health with a rich conceptual background.” The cultural context of loneliness, including its factors and features, has unique characteristics that necessitate context-specific interventions. As a recent scoping review suggests, there is a need to improve existing interventions that address the social isolation and loneliness of older adults by making them more context-specific aiming at “discerning what interventions work for specific subsets of this [older] population” (50).

Study results also highlight experiences of mistreatment and victimization among older people both in community and health care settings. “Although robust prevalence studies are sparse in low-income and middle-income countries, elder abuse seems to affect one in six older adults worldwide, which is roughly 141 million people” (51). Several reviews suggest that elder abuse is a serious but neglected problem worldwide (51–53), but with strong ethical implications (54). The findings of this review show that particular cultural beliefs that link witchcraft with old age are likely to be a reason for the mistreatment and victimization of older people in Eastern Africa. This appears inconsistent with findings from other studies (34, 35) that present traditional values as only beneficial and modernization as harmful to older people. Traditional cultural values in Eastern Africa, even though they are proven to maintain the respect and dignity of older people (34, 35), do not create an environment where older adults live without experiencing ethically questionable conditions. In some instances, traditional values are reported to result in the killing of older people in some parts of Eastern Africa. For example, in recent years, more than 500 older people have been murdered between 2005 and 2011 in Tanzania because of suspicions that they were witches and this number increased to 765 in 2013 only, of which 505 were women (55). This is neither a recent phenomenon nor a belief limited to (Eastern) African communities. Many innocent older people have been killed in the name of witchcraft and sorcery in early modern Europe (56, 57). Elder mistreatment and their victimization have also a gender aspect in that older women are more vulnerable to such experiences than older men (55, 58). In fact, it is a global challenge that women pass through unfavorable health-related experiences on the sole basis of gender (58).

Additionally, elder mistreatment stands out as a problem in health care settings as well and corroborates research from other locations that highlight health professionals' negative attitudes and mistreatment of older patients (59, 60). In line with this, studies from other African countries such as South Africa that are located outside of the Eastern region suggest that mistreatment of older patients could happen in health care facilities (61). Elder mistreatment in health care settings could be linked to the lack of geriatric knowledge and skills among health professionals. Geriatric knowledge is often reported as a very important factor in determining the quality of elder care (60, 62, 63).

Inaccessibility of health care by older adults is the most discussed theme in our findings, which has implications for access to care debates in several ways. Health care access is one of the main challenges for ensuring global healthy aging (64). The problem persists in both strong (65, 66) and fragile (67, 68) health systems. As the findings of this review show, resource limitation exists on both sides–the service receiver and provider, thereby making the problem challenging to address. On one side, governments lack the resources to make health facilities easily available for older people and tailor health systems to meet the needs of older adults (10, 35). In this case, access to health care raises the question of distributive justice (69). As Galarneau (70) writes, “the issue of access to health care raises fundamental concerns about the complex institutional structuring of health care organization and finance, as well as the political processes of resource allocation and rationing.” On the other side, older adults as well-lack the resources to access health care (3, 45). The inability and struggle to manage out-of-pocket health expenditures create among older adults stress and feeling of burden (71, 72), which increase the risk of further health complications. Similar experience of lack of adequate health insurance coverage is reported in Western African countries such as Ghana and Senegal (73). Elaborating and lamenting the ethical implications of this unfavorable circle in the African context, Chuwa writes: “the vicious circle of poverty and disease, which dehumanize the poor, is doubly unethical” (74). As a global scoping review on aging and poverty reports, poverty and social exclusion are the two most significant barriers for older persons to take part in the development of society and to have an equal share of its benefits like other population groups (75).

Finally, inadequate commitment from authorities to ensure wellbeing among older adults was a problem, which is consistent with studies from other regions in Africa (76–78). In the context of health care facilities, this can be demonstrated through poor medication administration that further complicate the health conditions of older people. And more broadly, inadequate commitment from government authorities results in, *inter alia*, health inequality among older persons. Literature asserts that differences and inequalities in the life and health of older people–differences that are labeled “unnecessary, avoidable, unfair, and unjust” (79)–are mostly the results of social determinants such as socioeconomic conditions, working conditions and education (80, 81). The fair distribution of resources and opportunities would play an important role in mitigating such inequalities. The health care of older adults has implications for not only public health but also society in general. Elaborating this, the WHO states:

“Older people contribute to society in many ways–whether it be within their family, to their local community or to society more broadly. However, the extent of these human and social resources, and the opportunities available to each of us as we age, will be heavily dependent on one key characteristic: our health. … If these added years are dominated by declines in physical and mental capacities, the implications for older people and for society may be much more negative” (6).

## Limitations

One major limitation in this scoping review is that there is very little literature in comparison with many other regions (82, 83) and this made it difficult to draw strong claims about the results, highlighting an urgent need for more empirical work on the topic. Even though it was not possible to broadly discuss the ethical issues due to the limited availability of literature, the included articles provided useful information to address the overall aim of this scoping review. An additional limitation is that studies that include all African countries were excluded. This might have resulted in the exclusion of important articles that could bring more insight into the research inquiry. However, due to the considerable variation between socio-cultural contexts across the continent, including those studies might also have confounded the overall findings. Similarly, generalization of the study findings is limited as the data covered only 7 of the 18 countries in Eastern Africa. Finally, some of the countries included in this review may publish in languages other than English, which were not included in this review.

## Conclusion

This scoping review summarizes the ethical issues arising in relation to providing care for older persons in the African context. It has the potential to create greater awareness of the growing complexities of caregiving for older persons in today's rapidly evolving and globalizing world. Taking a whole-of-government approach, governments in Eastern Africa (and beyond) could engage different sectors from health, economy, education, and culture to device comprehensive solutions that can address the challenges of older adults at different levels. Concerning such a multi-sectoral approach, another helpful step is to mainstream aging at all levels possible, so that there will be increased awareness about aging and integrated efforts to tackle the challenges therein. Further research is needed to investigate specific ethical issues such as health care access and elder abuse arising in relation to old age care in Eastern Africa, as well as to inform global health efforts that aim at improving the lives of older persons, particularly in low- and middle-income countries.

## Data Availability Statement

The original contributions presented in the study are included in the article/Supplementary Material, further inquiries can be directed to the corresponding author.

## Author Contributions

KM and TW contributed to the designing and planning of the literature search and were involved in performing early stage screening of the search results. KM wrote the first draft, performed the literature search, and analyzed the included articles. TW provided inputs for literature search and included articles. KM, JS, BE, MK, and SM were involved in full-text reviewing of articles during the final stage of literature screening. All authors substantially contributed to the revision of the subsequent manuscript versions, read, and approved the final version of the manuscript.

## Funding

This study was jointly funded by the Institute for Biomedical Ethics, University of Basel, and the European Union's Horizon 2020 research and innovation program under the Marie Skłodowska-Curie grant agreement No 801076, through the SSPH+ Global PhD Fellowship Programme in Public Health Sciences (GlobalP3HS) of the Swiss School of Public Health.

## Conflict of Interest

The authors declare that the research was conducted in the absence of any commercial or financial relationships that could be construed as a potential conflict of interest.

## Publisher's Note

All claims expressed in this article are solely those of the authors and do not necessarily represent those of their affiliated organizations, or those of the publisher, the editors and the reviewers. Any product that may be evaluated in this article, or claim that may be made by its manufacturer, is not guaranteed or endorsed by the publisher.
