# Supplementary material for: Care of Older Persons in Eastern Africa: A Scoping Review of Ethical Issues
Source: Front Public Health. 2022 Jul 6;10:923097. doi: 10.3389/fpubh.2022.923097 (PMC9298985; doi:10.3389/fpubh.2022.923097)
Supplement: Supplementary file 1 [file Data_Sheet_1.pdf]

Supplementary Appendix A: Search strategies used for databases

1. **Search strategy used for CINAHL, Africa-Wide Information, AgeLine, MEDLINE, and SocINDEX**

(MH "aged+") OR (MH "geriatrics+") OR (MH "geriatric-nursing+") OR (TI elder* OR AB elder* OR TI "old-age" OR AB "old-age" OR TI "olderadult*" OR AB "olderadult*" OR TI "olderpatient*" OR AB "olderpatient*" OR TI "old*- people" OR AB "old*- people" OR TI "old*- person*" OR AB "old* person*" OR TI "olderpopulation" OR AB "older-population" OR TI "senior-citizen*" OR AB "senior-citizen*" OR AB "ag#ing-population" OR TI "ag#ing-population")

AND

(MH "Health Care Delivery+") OR (MH "Gerontologic Care") OR (MH "Home Health Aides") OR (MH "Tertiary Health Care") OR (MH "Patient Centered Care") OR (MH "Home Health Care+") OR (MH "Housing for the Elderly") OR (MH "Residential Care+") OR (MH "Primary Health Care") OR (MH "Medical Care") OR (MH "Long Term Care") OR (MH "Day Care") OR (MH "Patient Care+") OR (MH "Nursing Care+") OR (MH "Family Centered Care+") OR (MH "Nursing Homes+") OR (MH "Gerontologic Nursing+") OR (TI care* OR AB care* OR TI “geriatric care” OR AB “geriatric care” OR TI “informal care” OR AB “informal care”)

AND

TI “east* Africa” OR AB “east* Africa” OR TI Burundi* OR AB Burundi* OR TI Comoros* OR AB Comoros* OR TI Djibouti* OR AB Djibouti* OR TI Eritrea* OR AB Eritrea* OR TI Ethiopia* OR AB Ethiopia* OR TI Kenya* OR AB Kenya* OR TI Madagascar* OR AB Madagascar* OR TI Malawi* OR AB Malawi* OR TI Mauritius* OR AB Mauritius* OR TI Mozambique* OR AB Mozambique* OR TI Reunion* OR AB Reunion* TI Rwanda* OR AB Rwanda* OR TI Seychelles* OR AB Seychelles* OR TI Somalia* OR AB Somalia* OR TI Tanzania* OR AB Tanzania* OR TI Uganda* OR AB Uganda* OR TI Zambia* OR AB Zambia* OR TI Zimbabwe* OR AB Zimbabwe*

2. **Search strategy used for APA PsycInfo**

((east* and Africa) or Burundi* or Comoros* or Djibouti* or Eritrea* or Ethiopia* or Kenya* or Madagascar* or Malawi* or Mauritius* or Mozambique* or Reunion* or Rwanda* or Seychelles* or Somalia* or Tanzania* or Uganda* or Zambia* or Zimbabwe*).ti,ab.

AND

((primary and health and care) or (long and term and care) or (home and care) or (patient and care) or (geriatric and care) or (nursing and care) or (informal and care) or (family and care) or (resident* and care) or (assisted and living) or care or caregiving).ti,ab.

AND

((geriatr* and patient*) or aged or geriatr* or elder* or (old* and age) or (older and adult*) or (older and patient*) or (old* and people) or (old* and patient*) or (old* and person*) or (old* and population) or (senior and citizen*) or (ageing and population) or (aging and population) or ageing or aging).ti,ab.
