# Supplementary material for: Care of Older Persons in Eastern Africa: A Scoping Review of Ethical Issues
Source: Front Public Health. 2022 Jul 6;10:923097. doi: 10.3389/fpubh.2022.923097 (PMC9298985; doi:10.3389/fpubh.2022.923097)
Supplement: Supplementary file 2 [file Table_1.pdf]

Supplementary Appendix B: Justification for the ethical relevance of themes

Themes	Bioethical concepts to which the theme is connected	Explanation as for why the theme was deemed an ethical issue
Loneliness and isolation	Fairness	In our analysis, this theme was linked to the question of whether it is fair for older persons to lose their traditional supports and company in a context where communal living is the normal trend. Additionally, loneliness has negative impact on human health and it is unfair for older persons to experience this without their choice.
Mistreatment and victimisation	Non-maleficence, justice, fairness	Mistreatment and victimisation inflict harm upon the victim, hence a critical ethical concern. In this theme, it is shown that older persons were verbally and physically abused in both health care and community settings and these acts are ethically unacceptable.
Inaccessibility of health care services	Justice, fairness, beneficence, health inequality	Health care access is one of the highly discussed topics in the field of health care ethics. In this theme, it was illustrated that older adults have limited access to health care due to factors such as inadequate availability of geriatric services and knowledge, health facilities being located in places inconvenient for older patients to reach, and financial constraints to cover health service costs. This implies that health services are not tailored to the health care needs of older persons, which could result in worsening health conditions among older patients.
Medical errors	Beneficence, non-maleficence, autonomy	This theme was framed as an ethical issue since medication error results in several ethical concerns such as harm to patients, quality of care, disclosure of mistakes to patients, and patient trust.
Lack of government attention to older persons	Justice, fairness	This theme discusses the inadequate commitment from government authorities to address the needs of older people. For example, older persons are not included in existing social security schemes and there is limited legal support for older persons who report abuse. These challenges are critical ethical concerns as they imply unfairness and lack of distributive justice.
Gender inequalities in old age	Fairness, justice, health inequality	This theme relates to bioethical concepts such as fairness, justice and health inequality. As illustrated in the theme, older women receive unfair treatment and have limited access to health care on the sole basis of their gender.
